# Alleviation of the adverse effects of NaCl stress on tomato seedlings (*Solanum lycopersicum* L.) by *Trichoderma viride* through the antioxidative defense system

**DOI:** 10.1186/s40529-023-00368-x

**Published:** 2023-02-09

**Authors:** Rabab A. Metwally, Shereen A. Soliman

**Affiliations:** Botany and Microbiology Department, Faculty of Science, Zagazig University, Zagazig, 44519 Egypt

**Keywords:** Antioxidant capacity, H_2_O_2_ content, Plant growth, Proline content, Reactive oxygen species, Seedling growth, Salt stress

## Abstract

**Background:**

*Trichoderma viride* are well known for their biocontrol capabilities, but little is known about how they stimulate plant development and increase their resistance to salt stress. One of the main abiotic factors limiting crop development and yield is salt stress. Therefore, the purpose of this work was to ascertain how NaCl effects on *T. viride* growth as well as on the seedlings morphological and physio-biochemical parameters of tomato (*Solanum lycopersicum* L.) under plate culture conditions. Additionally, a pot experiment was conducted to determine how *T. viride* affected the development characteristics of tomato plants subjected to various salt concentrations (50 and 100 mM NaCl). *T. viride's* contribution to tomato seedling stress tolerance was also closely examined.

**Results:**

Results showed that 100 mM NaCl decreased the colony diameter of *T. viride* by 13.4% compared to the control. Under plate and greenhouse conditions, tomato seedlings exposed to salt exposure exhibited an overall decline in growth. Also, a reduction in relative water content (RWC) and protein contents occurred under salt stress. At the same time, increases were found in proline, total phenolics, flavonoids, H_2_O_2_ content, malondialdehyde, likewise the activities of peroxidase (POD), catalase (CAT), polyphenol oxidase (PPO), and ascorbate peroxidase (APX) enzymes. Even though, with *T. viride* application, the salt negative effects on both morphological and physio-biochemical parameters were mitigated to a greater extent. *T. viride* increased proline and total antioxidant capacity (TAC) in tomato seedlings at 100 mM NaCl by an average of 20.66 and 43.82% compared to their comparable control. *T. viride* increased the activities of CAT, PPO, and APX enzymes by 74.6, 58.48, and 61.61% at 50 mM NaCl compared to non-saline control seedlings. As well, *T. viride* decreased MDA and H_2_O_2_ contents by an average of 14 and 24.8% in tomato seedlings at 50 mM NaCl compared to their comparable control. Also, under 100 mM NaCl, the *T. viride*-treated tomato seedlings showed increased total phenolics (17.85%) and flavonoids (33.17%) compared to non- treated one.

**Conclusion:**

Hence, our research sheds new insight on the pathways by which *T. viride* can boost tomato seedling tolerance to salt stress at morphological and physio-biochemical levels by activating both enzymatic and non-enzymatic antioxidant defense systems.

## Introduction

Due to their sessile nature, plants are frequently exposed to various biotic and abiotic stresses that can negatively impact agriculture productivity by up to 50%. These stresses are interrelated and can cause biochemical, morphological, and physiological changes that can lead to plant death (Evelin et al. 2019; Gupta et al. 2020; Soliman et al. 2022). After potatoes, tomatoes (*Solanum lycopersicum* L.) are the second-most important solanaceous crop produced and consumed globally (Al-Aysh et al. 2012; Metwally et al. 2022a), with a global production of 186.8 million tons in 2020 (FAO 2020). It is nutrient-rich and includes 37 minerals, water, proteins, fibers, carbohydrates, calories, and vitamins (Ngezahayo et al. 2019). It serves as an antioxidant, lowering blood pressure, preventing cancer and eye illnesses, and decreasing the likelihood of kidney stone formation (Tanveer et al. 2020). In Egypt, tomato is cultivated on a total area of 170.862 hectares (FAO 2019). This demonstrates the necessity of raising this crop's national production. However, abiotic stresses are still present during tomato growth despite the use of resistant varieties, and these pressures can occasionally result in significant damage (Tanveer et al. 2020).

Salinity is one of these abiotic stressors that prevents plants from growing (Abdelhameed and Metwally 2018; Dief et al. 2021; Abdelhameed et al. 2021; Abdalla et al. 2022). Universally and also in Egypt, large areas of land were out of cultivation due to salt accumulation (Moghazy and Kaluarachchi 2020). Recently, according to the Russian classification of the East Nile Delta area, soil salinization levels were 71% non-saline, 10.5% mild saline, 9% moderate saline, 3.8% strong saline, and 5.7% very strong saline (Hammam and Mohamed 2020). Plants undergo dehydration, nutritional disorders, oxidative stress, membrane instability, and decreases in metabolic and photosynthetic activity in saline soil (Metwally and Abdelhameed 2018; Zhang et al. 2019a; Abdelhameed et al. 2021). Salinity also inhibits the intake of water and causes a hazardous accumulation of Na^+^ and Cl^−^ in the cytosol and other cell organelles (Tavakkoli et al. 2010; Abdalla et al. 2022). To lessen the detrimental consequences of salt stress, significant efforts have been made to develop salt-tolerant plant genotypes by conventional breeding or genetic engineering. However, these attempts have shown limited success due to transgenic plants’ ease of losing the functional genes necessary for salt tolerance (Glick 2007).

An alternate technique for increasing plant tolerance to salt stress is to use microorganisms that promote plant growth. Arbuscular mycorrhizal fungi are one of these growth-promoting microorganisms that have been shown to enhance the capacity of plants like fenugreek and cowpea to withstand salt (Metwally and Abdelhameed 2018). Additionally, Trichoderma species can offer opportunistic symbionts to help plants become more resilient to abiotic stressors (Mastouri et al. 2010; Metwally 2020). These fungal species are often found in the rhizosphere and can provide beneficial effects on plant growth and yields (Metwally and Al-Amri 2019; Zhang et al. 2019b; Metwally et al. 2021; Abdelhameed and Metwally 2022). The ameliorative effects of *T. harzianum* (Th-6) on maize and rice below a hydroponic saline environment were examined (Yasmeen and Siddiqui 2018). Additionally, *T. harzianum* bio-priming of wheat seeds reduced the deleterious effects of salinity stress (Rawat et al. 2011). Although the exact mechanism by which *Trichoderma* spp. promotes plant development under salt stress is unknown, numerous observations with various species revealed that some metabolic processes and pathways may be involved (Contreras-Cornejo et al. 2014; Yasmeen and Siddiqui 2018). For instance, *T. virens* as well as *T. atroviride* enriched *Arabidopsis* seedlings growth via improved Na^+^ rejection, osmolyte synthesis, and root growth (Contreras-Cornejo et al. 2014). Similar to this, *T. asperellum* altered the levels of phytohormones and the capacity for phosphate solubilization in the cucumber plant to minimize the effect of salt stress' destructive effects (Zhao and Zhang 2015). Additionally, *T. longibrachiatum* T6 improved the anti-oxidative defense [peroxidase (POD), and catalase (CAT)] to increase wheat tolerance to NaCl stress (Zhang et al. 2016). Additionally, *T. harzianum* increased the amount of antioxidant enzymes in Indian mustard roots (Ahmad et al. 2015). Despite these various researches, there is still not enough knowledge to fully comprehend how salt stress affects plants.

In a previous study, we isolated *T. viride* strain from soil rhizosphere of the El-Sharkia governorate (Metwally and Al-Amri 2019). Our previous research showed that *T. viride* had a higher potential to enhance the onion plant growth under controlled conditions. However, our previous study was unable to examine whether this fungal strain can enhance plant tolerance under salt stress, and little is known about whether this function of the *T. viride* can be retained under various levels of salt stress. Therefore, the current study was carried out to investigate some morphological and physio-biochemical characteristics of tomato seedlings exposed to salinity as well as to explore the potential mechanism of *T. viride* in improving and mitigating induced by salt stress, which is used to find a salinity remedy that is both practical and efficient.

## Materials and methods

Experiments were carried out at the Laboratory of Mycology, Faculty of Science, Zagazig University, Egypt. In the studies outlined below, each experiment was carried out twice over a period of time with five replicates of each treatment.

### Fungal, plant material, and salt treatment

#### Fungal material

*Trichoderma viride* strain RA1 was previously obtained from the soil rhizosphere of the El-Sharkia governorate (Metwally and Al-Amri 2019) and recently molecularly identified and deposited in NCBI GenBank under the accession number ON479613 (Abdelhameed and Metwally 2022). *T. viride* was cultured on potato dextrose-agar (PDA) medium (Sigma-Aldrich, St. Louis, MO, USA) at 28 °C for 7 days until sporulation.

#### Plant material, salt treatment, and growth under plate culture conditions

The tomato (*Solanum lycopersicum* L.; Tomato HYBRID Seven F.1) was purchased at the Minia Al-Qamh, El-Sharkia Governorate, local market. Tomato seeds with a uniform shape and size were germinated in the laboratory at 25 ± 1 °C in the dark in October 2020 on sterile filter paper for 4 days till the emergence of the radicle of 0.6 cm in length. Sodium hypochlorite (7% NaOCl) was used to surface sterilize similar germinated seeds for 8 min. After disinfection, all the germinated seeds were rinsed with sterile distilled water 5 times, left for dryness on sterilized filter paper, and then transferred under aseptic conditions on agar plates containing MS medium (Murashige and Skoog basal salts mixture, Sigma-Aldrich). Six mm diameter of actively growing fungal agar discs of *T. viride* was inoculated in the opposite ends of 4-day-old germinated tomato seedlings (10 seedlings/plate) on agar plates (MS medium with 50 and 100 mM NaCl or without salt as control). Table 1 showed the details of the study treatments. In order to allow root growth along the agar surface and unhindered aerial growth of the hypocotyls, plates were positioned vertically at a 65° angle. Plates were then left for growth at 25 °C in a laboratory with a photoperiod of 16 h of light and 8 h of darkness. After 4 and 6 days of planting tomato seedlings on MS medium, the NaCl stress effects on the appearance of the first two cotyledonary leaves were recorded in Table 3. Moreover, 10 days after salt treatment, tomato seedlings were randomly collected and kept for further analysis.

**Table 1:** Different treatments

T_0_	T_1_	T_2_	T_3_	T_4_	T_5_
Control	*T. viride*	NaCl (50 mM)	NaCl (50 mM) + *T. viride*	NaCl (100 mM)	NaCl (100 mM) + *T. viride*

### Measurements

#### Effect of NaCl concentrations on the linear growth and mycelia dry weight of *T. viride*

*T. viride* mycelia discs (6 mm) of active culture were planted in sterile PDA media with salt concentrations of 50, 100, 150, 200, 250, and 300 mM NaCl and then cultured at 25 °C with supplemental day/night lighting of 16/8 h. The control was PDA media inoculated with T. viride mycelia disc but lacking NaCl (0 mM NaCl). The colony diameter was measured 2 and 4 days after inoculation. The morphological deformations caused by NaCl concentrations on the mycelia of *T. viride* on PDA plates were directly examined. Hyphal threads at the end of the fungus colony were taken out, checked for abnormalities, and compared to the control under a light microscope (Leitz WETZLAR, Germany).

The potato dextrose broth media were inoculated with 0.5 mL of *T. viride* (1 × 10^8^ spores mL^−1^) spore suspension in 250 mL flasks with 100 mL each. The media were dispersed with varied NaCl concentrations (50, 100, 150, 200, 250, and 300 mM). Media without NaCl solution were considered as the control (0 mM NaCl). Flasks were incubated at 25 °C for 5 days with shaking at 180 rpm/minute. On day 5, the fermentation was filtered three times through sterilized filters before the mycelia were collected, dried in an oven for three hours at 80 °C, and weighed to determine their dry weight.

#### Seedlings growth assessment

Ten days after being treated with NaCl, tomato seedlings were harvested. Both the fresh weight (FW) and height (cm) of the seedlings were assessed. All samples of tomato seedlings were oven dried at 60 °C for 48 h to obtain a constant weight for determining the dry weight (DW). Moreover, the following formula was used to determine the seedling height stress index (SHSI):

$${\text{SHSI}} = \frac{{\text{Height of stressed seedlings}}}{{\text{Height of control seedlings }}} \,{\times}\, 100$$

#### Evaluating the water status of seedling

Tomato seedlings were tested for their water content (WC), relative water content (RWC), and water saturation deficit (WSD) (Barr and Weatherley 1962). To measure FW, tomato seedlings were divided into small parts. Segments of seedlings were floated in deionized water for 4 h under light and ambient temperature. Surface water was removed after 4 h, and samples were weighted once more to get a fully turgid weight (TW). The samples were weighed again after being dried in a 60 °C oven for 24 h to determine the DW, RWC, and WSD using the following equations:

$${\text{RWC }}\left( {{\% }} \right){ } = \frac{{\left( {{\text{FW }}{-}{\text{ DW}}} \right){ }}}{{{ }\left( {{\text{TW }}{-}{\text{ DW}}} \right){ }}}\,{\times}\,100$$

$${\text{WC }}\left( {{\% }} \right){ } = \frac{{\left( {{\text{FW }}{-}{\text{ DW}}} \right){ }}}{{\text{ FW }}}\,{\times}\,100$$

$${\text{WSD }}\left( {{\% }} \right) = { }100 - {\text{RWC}}$$

*FW and DW stand for fresh and dry weight, respectively.

#### Lipid peroxidation and H_2_O_2_ assay in tomato seedling

MDA accumulation in tomato seedlings treated or not with *T. viride* under 0, 50, and 100 mM NaCl was measured (Ohkawa et al. 1979). In a brief, 3 mL of 5% trichloroacetic acid (TCA) was used to grind up known tomato seedlings, and the homogenate was then centrifuged for 10 min at 6000 rpm. A sample of 2 mL of the supernatant was mixed with 2 mL of 20% trichloroacetic acid (TCA) containing 0.5% thiobarbituric acid (TBA) for 25 min at 96 °C and then cooled quickly on an ice bath. In order to measure the absorbance at 532 nm, a UV–visible spectrophotometer called RIGOL (Model Ultra-3660) was used. µmol g^−1^ FW was used to express the MDA content.

As well, H_2_O_2_ content in a known FW of tomato seedlings was determined (Alexieva et al. 2001) by homogenizing with 3 mL of 0.1% TCA. A known volume of the homogenate (0.5 mL) was added together with 2 mL of 1 M potassium iodide (KI) and 0.5 mL of potassium phosphate buffer (100 mM, pH 7). The reaction mixture was centrifuged for 10 min after being left in the dark and at room temperature for 1 h. By measuring the absorbance at 390 nm and expressing the result as µg g^−1^ of FW, the H_2_O_2_ content was determined.

#### Total phenolic content in tomato seedlings

After 95% ethanol extraction, the total phenolic content of tomato seedlings grown under control conditions or salt stress conditions and treated with *T. viride* was evaluated (Jindal and Singh 1975). To 1.4 mL of distilled water and 0.1 mL of 50% Folin-Ciocalteu phenol reagent, 0.5 mL of the extract was added. Sodium carbonate (0.4%) was then added to the sample. After 2 h, the resulting mixture was maintained, and the absorbance at 650 nm was measured. The total phenolic content of tomato seedlings was quantified as mg/g FW of gallic acid equivalent (GAE) using gallic acid as the standard.

#### Total flavonoids content in tomato seedlings

After grinding a known FW with 95% ethanol, the total flavonoid content of the tomato seedlings was determined by the AlCl_3_ colorimetric technique (Zou et al. 2004) using a quercetin standard curve and it was expressed as mg/g FW of quercetin equivalent (QE). After centrifuging the mixture, the supernatant was gathered. Around 5 mL of distilled water was added to the supernatant followed by the addition of 0.7 mL of 5% NaNO_3_ and 0.6 mL of 10% AlCl_3_. Following the addition of 3 mL of 1 M NaOH and 2.5 mL of distilled water, the solution was again left for 1 min. At 510 nm, the absorbance was measured in comparison to a blank.

#### Proline contents in tomato seedling

Proline content in a known tomato seedlings FW (Bates et al. 1973) was assessed by homogenizing in 5 mL of 3% aqueous sulphosalicylic acid. After that, the reaction was then quickly stopped in an ice bath after being combined with 2 mL of supernatant, 2 mL of glacial acetic acid, and 2 mL of acid ninhydrin at 100 °C for 1 h. The reaction mixture was then given 4 mL of toluene. From the aqueous phase, the toluene-containing chromophore was aspirated, and the absorbance was measured at 520 nm. Using a standard curve, the proline concentration was computed as follows:

$${\text{Proline}}\,{\text{concentration}}\,\left( {{\raise0.7ex\hbox{${{\mu g}}$} \!\mathord{\left/ {\vphantom {{{\mu g}} {\text{g}}}}\right.\kern-0pt} \!\lower0.7ex\hbox{${\text{g}}$}}\,{\text{FW}}} \right)\, = \,\frac{{\left( {{\raise0.7ex\hbox{${{\mu g}\,{\text{proline}}}$} \!\mathord{\left/ {\vphantom {{{\mu g}\,{\text{proline}}} {{\text{mL}}}}}\right.\kern-0pt} \!\lower0.7ex\hbox{${{\text{mL}}}$}}\, \times \,{\text{mL}}\,{\text{toluene}}} \right)}}{{115.5\, \times \,{\text{g}}\,{\text{FW}}\,{\text{of}}\,{\text{sample}}}}$$

*115.5 is the molecular weight of proline.

#### Protein and ROS scavenging enzymes extraction and determination

After 10 days of NaCl treatment, total soluble proteins and antioxidant enzyme activities were measured using a known FW of tomato seedlings by homogenizing the sample with 5 ml of an extraction buffer containing 1 mM ethylenediaminetetraacetic acid (EDTA) and 50 mM k-phosphate buffer (pH 7). Extracts were utilized to evaluate the ROS-scavenging scavenging enzyme activities after being centrifuged at 10,000 rpm for 15 min. Using pyrogallol as the substrate at 470 nm, peroxidase (POD) activity was measured in accordance with the method of Chance and Maehly (1955). According to Aebi's (1983) approach, catalase activity was assessed by calculating the decline disintegration of H_2_O_2_ in absorbance at 240 nm. According to Beyer and Fridovich (1987) and Nakano and Asada (1981), the ascorbate peroxidase (APX) and polyphenol oxidase (PPO) activities in the tomato seedling extract were measured at 430 nm and 290 nm, respectively, and their activities were expressed as U g^−1^ FW. Also, the concentration of protein in the extracts was measured by Lowry et al. (1951).

#### Assay of total antioxidant capacity (TAC) in tomato seedlings

According to Prieto et al. (1999), the TAC in an aliquot of 0.5 mL of the tomato seedling extracts was assessed by adding 4.5 mL of reagent solution (0.6 M sulfuric acid, 28 mM sodium phosphate, and 4 mM ammonium molybdate). The absorbance was measured at 695 nm after the tubes had been incubated in a boiling water bath at 95 °C for 90 min and then left to cool to room temperature. The antioxidant activity is expressed as the number of gram equivalent of ascorbic acid and the TAC was reported as μg ascorbic acid equivalents/g FW.

#### Evaluation of *T. viride* effects on tomato growth under greenhouse conditions

In a pot experiment conducted in a greenhouse of the Botany and Microbiology Department, Faculty of Science, Zagazig University with temperatures ranging from 30 to 35 °C and relative humidity from 60 to 85%, five replicates for each treatment, the effects of *T. viride* on the growth of tomato seedlings grown under salt stress conditions were also evaluated.

Experimental treatments were applied to 30 days old tomato seedlings and being cultivated in plastic pots with 2 kg of sterile clay soil. Two tomato seedlings were put into each pot after being originally soaked for 4 h in a fungal suspension of *T. viride*. Also, pot soils were drenched with 100 mL of the prepared fungal inoculum (*T. viride* treated seedlings) or equivalent tap water (non- *T. viride* treated tomato seedlings). After transplanting for 1 week, salt solutions were added. Tap water was used to water the control treatments. Salinity levels and the application of *T. viride* were two parameters that were entirely randomized for plastic pots. There were two *T. viride* groups and two salt concentrations (50 and 100 mM NaCl). The tomato samples from all treatments were collected after 20 days of salt treatment.

#### Determination of morphological parameters

After 20 days of salt addition, roots of tomato seedlings from *T. viride* treated and non-treated plants were cleaned to remove soil particles and shoot height, leaves number, root length, FW and DW of shoot and root were recorded after placing in an oven at 70 °C for 2 days.

### Statistical analysis

Using the SPSS software, one-way ANOVA was performed on the data (SPSS V16.0, SPSS, Inc., Chicago, IL, USA). The Duncan’s multiple range test was used to determine the effects of the treatment, and *p* < 0.05 was used to express significance.

## Results and discussion

### NaCl stress consequence on the colony diameter and mycelia weight of *T. viride*

**Fig. 1:**
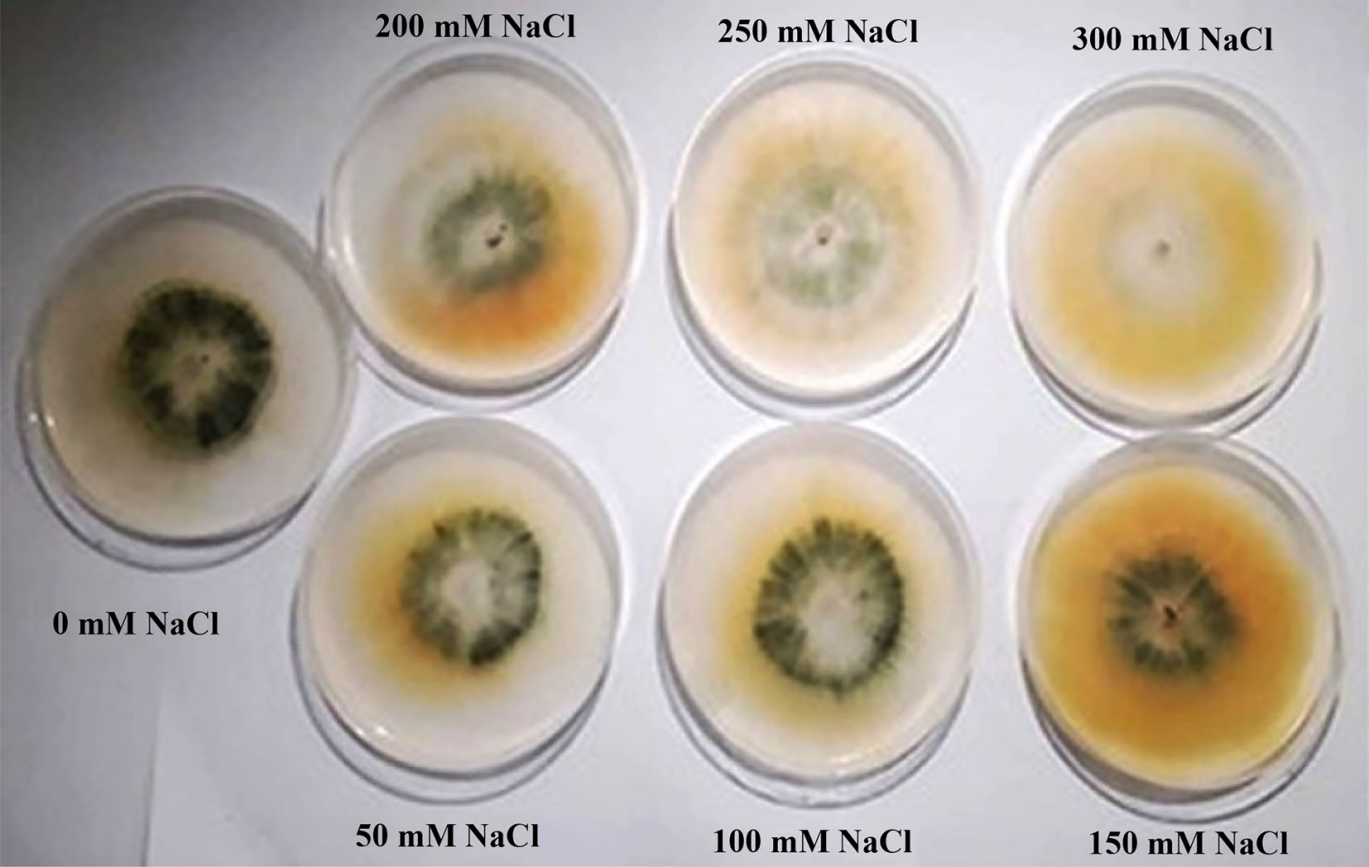
Illustrative photograph showing the effect of NaCl concentrations on *T. viride* growth colonies grown at 25 °C and photographed after 2 days. To determine the effects of NaCl on *T. viride*, mycelia discs (6 mm) of active culture were used to inoculate PDA medium with or without NaCl

**Fig. 2:**
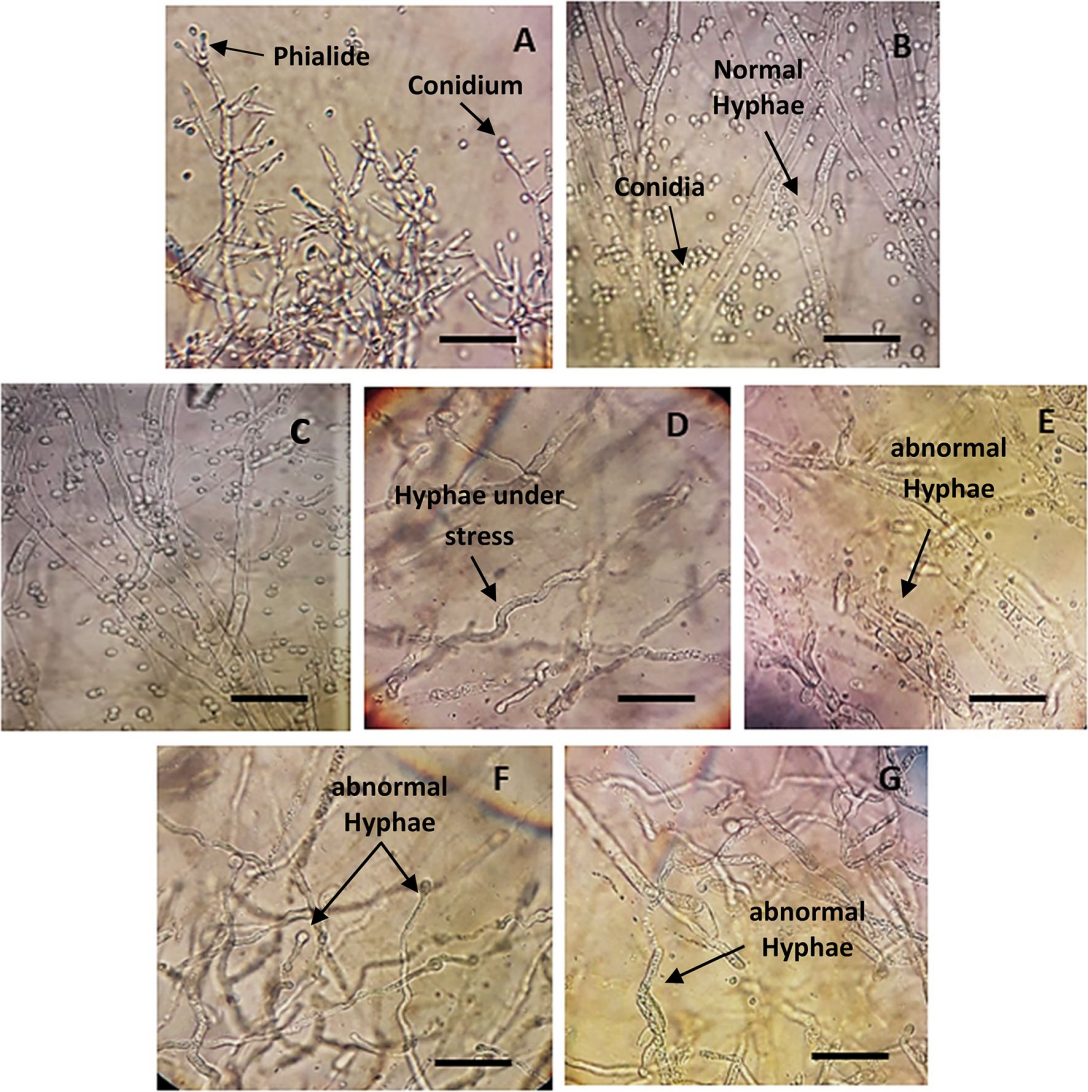
Effect of different salt concentrations (mM NaCl) on *T. viride* growth. **A** and **B** Normal hypha (Control), **C** *T. viride* hypha under (50 mM NaCl) stress, **D** and **E** *T. viride* hypha under (150 mM NaCl) stress, **F** and **G** *T. viride* hypha under (300 mM NaCl) stress. Scale bars: **A** = 20 µm. **B**–**C** = 30 µm. **D**–**G** = 25 µm

Plant growth is delayed by salinity, and the degree of this delay may depend on how the host, microbes, and salt level interact (Mastouri et al. 2010; Zhang et al. 2019a). Therefore, it is important to research how NaCl affects *T. viride* growth. According to our findings (Fig. 1 and Table 2), *T. viride'*s mycelia weight was more affected by low salt concentrations than the control after 5 days of incubation. Also, with increasing NaCl concentrations occurs a decrease in the dry weight of mycelia significantly (*p* < 0.05) (Table 1). Concerning *T. viride* colony diameter results, low salt concentrations (50 and 100 mM NaCl) cause a slight decrease in *T. viride* growth as shown in Fig. 1, however, the decrease is not significant. However, with higher salt concentrations, the differential inhibitory effects can be noticed (150, 200, 250, and 300 mM). This may be the effect of increasing the substrate's water potential, which inhibits the formation of fungal colonies at high salt concentrations (Zhang et al. 2019a). High salt levels may also influence intracellular proteins that may give extra osmotic potential to avoid plasmolysis, which is a metabolic process that occurs in the cytoplasm (Yeo 1983). Our results are compatible with Zhang et al. (2019b) with *T. longibrachiatum* T6. Furthermore, Contreras-Cornejo et al. (2014) observed that higher salt concentrations significantly reduced *T. atroviride* growth, but lower NaCl concentrations increased the colony diameter*.* This shows that the growth of *Trichoderma* spp. is dose-dependently affected by NaCl, with high salt limiting growth and low salinity boosting growth (Henk and Cuppers 1997). Furthermore, according to a study by Guo et al. (2018), 2% NaCl can stimulate *T. asperellum* development. Similarly, Rawat et al. (2012) stated that five of forty-five *T. harzianum* wild-type strains can grow and form spores in growth containing up to 240 mM NaCl. The optical microscopic examination reveals that the treatment of *T. viride* with high salt concentrations (150 and 200 mM NaCl) caused abnormal mycelial growth and considerable morphological changes, mainly manifesting as deformation, contraction, collapse, globular swellings occurred at the tips of hyphal strands and deformity of the conidium (Fig. 2D, E, F and G). In contrast, the mycelia of the control and that of low salt concentration (50 mM NaCl) are straight and well developed (Fig. 2A, B and C).

**Table 2:** Effect of NaCl concentrations on the linear growth and dry weight (g/100 mL) of *T. viride*

NaCl concentration	Colony diameter (cm) Days of incubation		DW (g/100 mL)
	2 days	4 days	
0 mM	5 ± 0.265a	8.1 ± 0.429a	0.5942 ± 0.0314c
50 mM	4.53 ± 0.239ab	7.11 ± 0.376ab	0.8718 ± 0.0461a
100 mM	4.33 ± 0.229abc	7.38 ± 0.391ab	0.7039 ± 0.037b
150 mM	4.18 ± 0.221bc	7.38 ± 0.391ab	0.497 ± 0.0263d
200 mM	3.83 ± 0.203bc	6.46 ± 0.342bc	0.342 ± 0.0181e
250 mM	3.65 ± 0.193cd	6.41 ± 0.339bc	0.3236 ± 0.0171e
300 mM	3.13 ± 0.165d	5.78 ± 0.306bc	0.2838 ± 0.015e

^*^Data are mean of 5 replicates ± standard error; the dry weight was determined 5 days after inoculation

Different letters in the same column mean significant difference at the *p* < 0.05 level by Duncan’s new multiple range test

### NaCl stress consequence on the appearance of cotyledonary leaves

One of the main environmental factors that restrict plant growth and causes biochemical changes in plants is high salinity, according to earlier studies of Mahmood et al. (2012) and Zhang et al. (2016). Our results show that salinity reduces the number of tomato seedlings that show the first two cotyledonary leaves after 6 days of salt application by 59.3 and 37.5% at 50 and 100 mM NaCl over their respective control ones. It means that salinity had negative effects on this parameter (Table 3). Additionally, salinity slows down ultimate germination over time relative to control by reducing the water potential of the germination substrate and lengthening the time it takes for seeds to germinate (Ayers 1952; Cuartero and Ferandez-Munoz 1998). Similar findings were made by Tanveer et al. (2020), who discovered that salinity reduced the proportion of seeds that germinated because it causes low water and nutrient uptake, which has an impact on seedling germination and development (AL-Mutawa 2003; Rawat et al. 2013; Abdulla et al. 2022).

**Table 3:** Effect of NaCl stress on the appearance of the first two cotyledonary leaves of tomato seedlings

Treatments	Number of seedlings		Percentage = Seedling number showing the first two cotyledonary leaves/total seedling number*100	
	After 4 days	After 6 days	After 4 days	After 6 days
T_0_	3 ± 0.587a	8 ± 0.577a	37.5	100
T_2_	2 ± 0.577a	4.33 ± 0.88b	25	59.3
T_4_	1.33 ± 0.66a	3.33 ± 0.667b	12.5	37.5

T_0_, T_2_ and T_4_ represent tomato seedlings grown on MS medium under 0, 50 and 100 mM NaCl stress

^*^Data are mean of 5 replicates ± standard error

Different letters in the same column mean significant difference at the *p* < 0.05 level by Duncan’s new multiple range test

### Plant growth promotion in *T.viride*-treated tomato seedlings under plate conditions

**Fig. 3:**
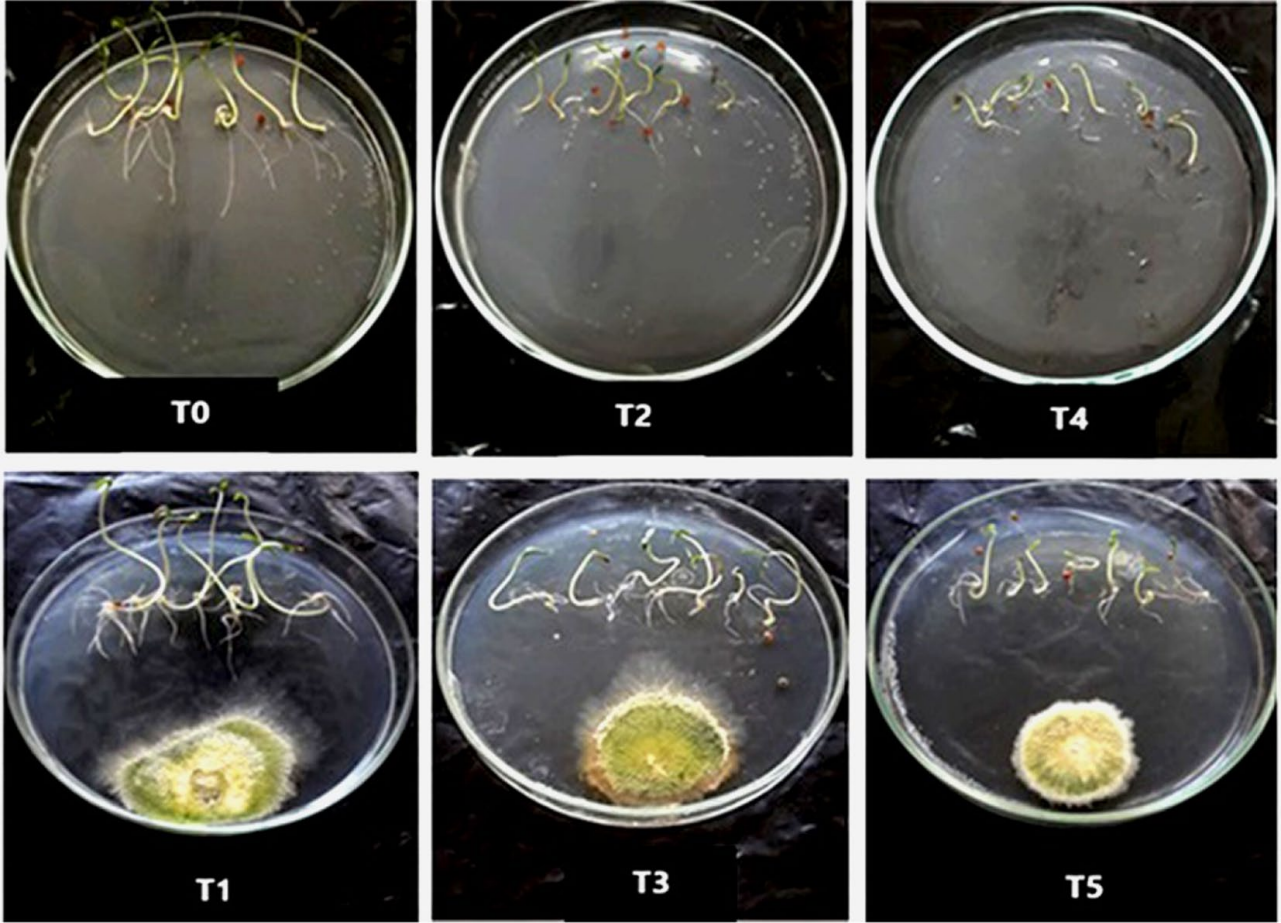
The phenology of control and NaCl stressed tomato seedlings under *T. viride* fungal application. T_0_, T_2_ and T_4_ represent tomato seedlings grown on MS medium under 0, 50 and 100 mM NaCl stress, while T_1_, T_3_ and T_5_ represent tomato seedlings grown on MS medium with *T. viride* under 0, 50 and 100 mM NaCl stress

**Fig. 4:**
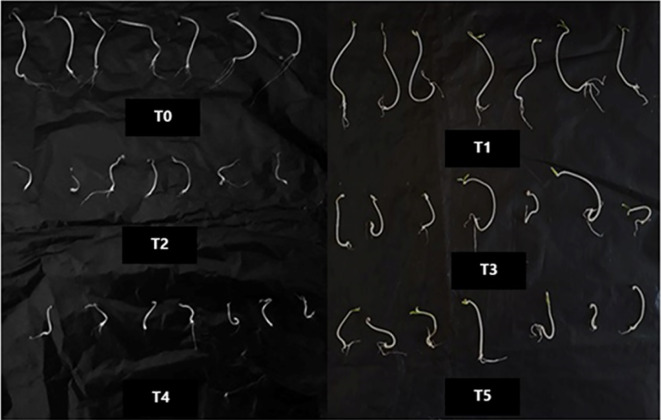
Photographs of tomato seedlings showing the differences between control and salt stressed seedlings under *T. viride* fungal application. T_0_, T_2_ and T_4_ represent tomato seedlings grown on MS medium under 0, 50 and 100 mM NaCl stress, while T_1_, T_3_ and T_5_ represent tomato seedlings grown on MS medium with *T. viride* under 0, 50 and 100 mM NaCl stress

**Fig. 5:**
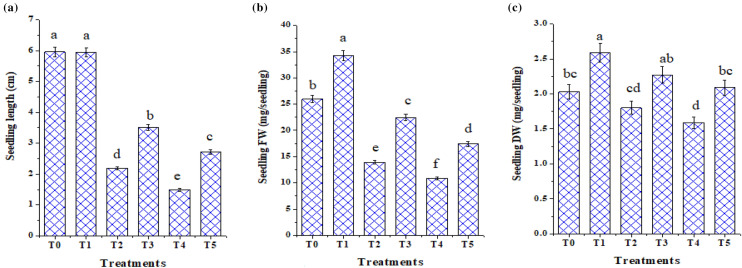
Effect of *T. viride* on seedling height, fresh (FW) and dry weights (DW) of tomato seedlings in response to salinity stress. T_0_, T_2_ and T_4_ represent tomato seedlings grown on MS medium under 0, 50 and 100 mM NaCl stress, while T_1_, T_3_ and T_5_ represent tomato seedlings grown on MS medium with *T. viride* under 0, 50 and 100 mM NaCl stress*.* * Data are mean of 5 replicates ± standard error. Different letters above bars indicate a significant difference between treatments using ANOVA followed by Duncan’s multiple range test (*p* < 0.05)

To assess the growth-promoting effects of *T. viride* on tomato seedlings under salt stress, measurements of seedling height, fresh weight (FW), and dry weight (DW) of tomato seedlings were made 10 days after NaCl application (Figs. 3, 4 and 5). The seedling FW and DW lowered by 33 and 26% and 15 and 19%, respectively, under 50 and 100 mM NaCl, over their respective control ones, according to our data, which demonstrate that these growth parameters were significantly (*p* < 0.05) reduced with NaCl treatment. Salt stress inhibitory effect was consistent with the findings of Metwally and Abdelhameed (2018), Zhang et al. (2016), and Dief et al. (2021) in fenugreek and wheat. The non-availability of mineral nutrients and the energy expended to minimize the negative effects of NaCl may be the reasons (Hashem et al. 2015). Additionally, it is recognized that osmotic stress, ion toxicity, and oxidative stress imposed by salt stress can all delay growth (Abdel Latef and Chaoxing 2014). However, the effect of salt was alleviated substantially with *T. viride* application, indicating that *T. viride* improves these measured variables significantly (Fig. 5). Our results are coherent with Metwally (2019) and Metwally and Al-Amri (2020) results about the improving capabilities of *T. viride* on onion growth and physiology. Likewise, Zhang et al. (2016) observed that NaCl inhibited wheat seedling growth and that this inhibitory effect was alleviated by adding *T. longibrachiatum* T6. Tomato seedlings treated with *T. viride* at 50 and 100 mM NaCl improved in height by 15 and 34% in comparison to NaCl-stressed stressed seedlings, respectively (Fig. 5), where *T. viride* reached the maximum seedling height in both control and NaCl treatments. The morphological traits of control or NaCl-stressed tomato seedlings treated with *T. viride* fungal application are shown in Fig. 4.

Related studies found that *T. harzianum* improved cucumber seedling growth following salt application (Zhang et al. 2019a). Additionally, numerous earlier investigations have demonstrated that *Trichoderma* sp. forms symbiotic interactions with a wide range of host plant roots and encourages those organisms' growth and development (Gupta et al. 2020). Trichoderma sp. is also known to produce a variety of antibiotics, including polyketides, trichodermol, peptaiboils, and steroids that aid in plant development (Harman et al. 2012). Additionally, according to Zhang et al. (2019b), the enhancement of plant development in saline conditions is highly dependent on the rise in ACC-deaminase activity and IAA synthesis in Trichoderma.

### Seedling height stress index (SHSI)

The seedling height stress index was reduced under both salt concentrations as compared to the non-saline MS medium (Table 4). However, *T. viride* increased the seedling height stress index (SHSI) under both salt concentrations with 58.98 and 45.55, respectively. The lowest SHSI was detected in tomato seedlings subjected to 100 mM NaCl. Furthermore, according to AL-Mutawa (2003) and Rawat et al. (2013), excessive salt concentrations cause reduced water and nutrient uptake, which has an impact on seedling germination and development. However, tomato seedlings treated with *T. viride* had a significantly higher percentage of SHSI under both salt concentrations. As well, *Trichoderma* symbiotic colonization has been demonstrated by Rawat et al. (2013) and Zhang et al. (2016) to accelerate root growth and cause the solubilization and sequestration of inorganic nutrients, which may be the mechanism for enhanced tolerance to osmotic stresses.

**Table 4:** Influences of NaCl stress and *T. viride* on seedling height stress index (%)** (**SHSI) and the water status of tomato seedlings

Treatments	Seedling height stress index (%) (SHSI)	Seedling water status		
		Water content (%) (WC)	Relative water content (%) (RWC)	Water saturation deficit (%) (WSD)
T_0_	–	92.71 ± 4.906a	91.56 ± 4.845ab	8.44 ± 0.446d
T_1_	–	93.31 ± 4.937a	96.54 ± 5.108a	3.46 ± 0.183e
T_2_	36.69	89.49 ± 4.735a	85.16 ± 4.526ab	14.84 ± 0.785b
T_3_	58.98	91.53 ± 4.843a	87.64 ± 4.638ab	12.36 ± 0.634c
T_4_	24.94	88.61 ± 4.688a	79.85 ± 4.225b	20.15 ± 1.066a
T_5_	45.55	93.66 ± 4.956a	84.58 ± 4.476ab	15.42 ± 0.816b

T_0_, T_2_ and T_4_ represent tomato seedlings grown on MS medium under 0, 50 and 100 mM NaCl stress, while T_1_, T_3_ and T_5_ represent tomato seedlings grown on MS medium with *T. viride* under 0, 50 and 100 mM NaCl stress

^*^Data are mean of 5 replicates ± standard error

Different letters in the same column mean significant difference at the *p* < 0.05 level by Duncan’s new multiple range test

### Seedling water status

Regarding relative water content (RWC), our results showed that tomato seedlings have a maximum RWC with *T. viride* under non-saline MS medium and minimum for seedlings exposed to 100 mM NaCl (Table 4). RWC of tomato seedlings exposed to 50 and 100 mM NaCl decreased by 7.00 and 12.78% compared to control ones grown under non-saline MS medium. Even though, 50 and 100 mM NaCl reduce RWC and WC of tomato seedlings as compared to control; this decrease is not significant. This is in accordance with Chaudhuri and Choudhuri (1997) and Metwally and Abdelhameed (2018) that salt stress affects the water status of jute and fenugreek. Physiological drought is a threat for plants growing in saline conditions because Na^+^ and Cl^−^ ions bind water which is essential for plant growth (Fuzy et al. 2008; Metwally and Abdelhameed 2018). Of particular note, the inhibitory effect of salinity on tomato seedlings was mitigated to some extend by *T. viride* application. Furthermore, under salinity, WSD was substantially increased; however, these effects were diminished when *T. viride* was applied (Table 4); as *Trichoderma's* effects enable plants to more efficiently use water to maintain a lower CO_2_ concentration within cells.

### The consequence of NaCl and ***T. viride*** on H_2_O_2_ content and lipid peroxidation

**Fig. 6:**
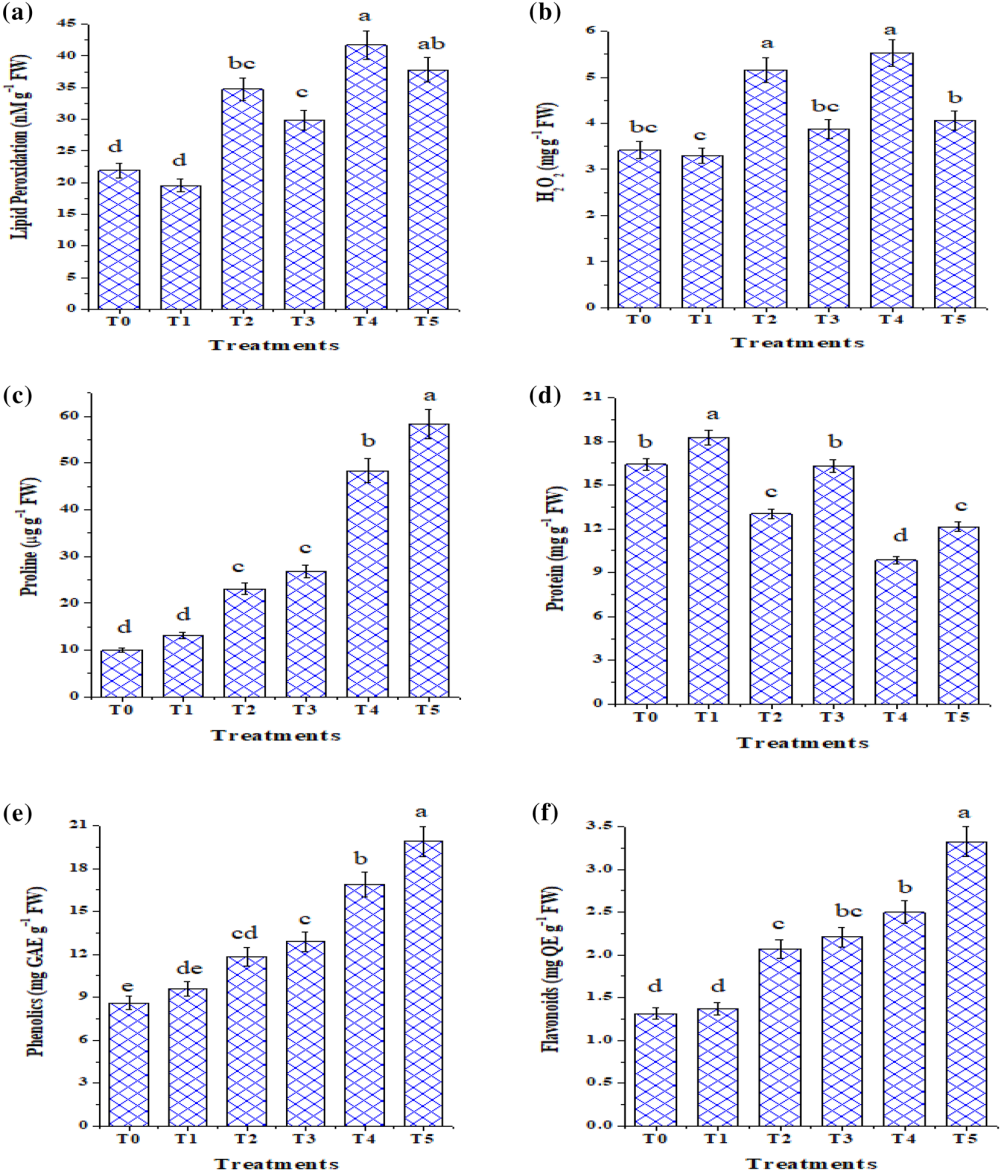
Effect of *T. viride* application on lipid peroxidation (nM/g FW), hydrogen peroxide (H_2_O_2_) content (mg/g FW), proline (μg/g FW), protein (mg/g FW), total phenolic (mg GAE/g FW) and flavonoid (mg QE/ g FW) contents of tomato seedlings in response to salinity stress. T_0_, T_2_ and T_4_ represent tomato seedlings grown on MS medium under 0, 50 and 100 mM NaCl stress, while T_1_, T_3_ and T_5_ represent tomato seedlings grown on MS medium with *T. viride* under 0, 50 and 100 mM NaCl stress*.* GAE: Gallic acid equivalent, QE: Quercetin equivalent *Data are the mean of five replicates ± standard error (n = 5). Different letters above bars indicate a significant difference between treatments using ANOVA followed by Duncan’s multiple range test (*p* < 0.05)

In consequence of superoxide radicals scavenging, H_2_O_2_ which is a toxic compound and injurious to plants is produced as a result of salt exposure. Higher concentrations of H_2_O_2_ in plants cause lipid peroxidation and membrane injury (Rawat et al. 2013; Zhang et al. 2016; Metwally et al. 2022b). Figure. 6a and b) shows that under 50 and 100 mM NaCl, tomato seedlings treated or not with *T. viride* had a substantial increase in H_2_O_2_ content. Significantly higher levels were maintained in control seedlings under both salinity levels (5.16 and 5.53 mg/g FW). However, *T. viride* treatment reduces H_2_O_2_ accumulation (Fig. 6b), where under salt stress; H_2_O_2_ content of control seedlings was significantly higher than that of *T. viride* treated seedlings. The minimum level of H_2_O_2_ was observed in seedlings treated with *T. viride* (3.30 mg/g FW) followed by non-treated ones (3.42 mg/g FW) grown under non-saline MS medium or control condition. Our results coincide with those of Rawat et al. (2013) and Zhang et al. (2019a), who used *T. harzianum* to treat chickpea and cucumber under salt stress conditions. The decreased levels of H_2_O_2_ in seedlings treated with *T. viride* demonstrate that these seedlings have a more efficient cellular free radical quenching system, which provides protection from oxidative stress.

Malondialdehyde (MDA), a product of lipid peroxidation, is frequently utilized as a marker for oxidative stress-induced free radical damage to cell membranes. Figure 6a shows our results regarding the effect of NaCl on MDA content in the presence and absence of *T. viride*. MDA significantly increased in tomato seedlings subjected to 50 and 100 mM NaCl by 58.3 and 90% relative to the control ones. These findings support Zhang et al. (2019a), who found that salt exposure induces the ROS produced in wheat seedlings to peroxide membrane lipids, which leads to the production of MDA (Metwally and Abdelhameed 2019; Abdelhameed et al. 2021). On the other hand, tomato seedlings with *T. viride* under salt stress were more effective than salt-stressed seedlings in lowering MDA contents, predicting membrane protection. Compared with their respective control, MDA content in tomato under control or 50 mM NaCl decreased by 11 and 14 percent with *T. viride* application. Our findings are consistent with Rawat et al. (2011) and Dief et al. (2021) that bio-priming wheat seeds with *T. harzianum* or *P. chrysosporium* decreased MDA accumulation.

### *T. viride* and NaCl effects on ROS scavenging in tomato seedlings

Low concentrations of ROS function as signaling molecules, but when they build up excessively, they can harm stressed plants. In turn, this can lead to oxidative stress, one of the harmful effects of plants exposed to environmental challenges (Nazar et al. 2011; Gupta et al. 2020; Nasrallah et al. 2020). These ROS can also impair the integrity of cellular membranes and enzyme activity (Metwally and Abdelhameed 2019; Abdelhameed et al. 2021). Plants rely on non-enzymatic and/or enzymatic processes for protection against the damage caused by oxidative stress. Additionally, Trichoderma sp. inoculation led to a variety of physiological improvements in plants, such as an increase in enzymatic and non-enzymatic antioxidants that strengthened plants' resilience to stress (Zhang et al. 2016).

### *T. viride* induces salt mitigation through a non-enzymatic mechanism (total soluble protein, proline, and total phenolic and flavonoid contents)

To assess whether *T. viride* induces salt tolerance in tomato seedlings due to a non-enzymatic mechanism, proline and soluble protein as substances capable of altering osmotic potentials (Fig. 6c and d) were detected. In order to minimize the negative effects of salt stress through balancing the solute potential, osmotic adjustment by reducing the osmotic potential plays a critical role in cellular water retention and turgor maintenance (Perez-Lopez et al. 2010), this in turn promotes cell growth.

Under stressful circumstances, proline stabilizes the membranes and prevents the breakdown of proteins and enzymes (Farooq et al. 2009). Regardless of the level of salt stress, we found that *T. viride* treatment in MS medium with tomato seedlings led to a significant (*p* < 0.05) increase in total soluble protein and proline levels. Following being exposed to NaCl, tomato soluble protein concentration dropped significantly (% of decreases were 20.58 and 40 after exposure to 50 and 100 mM NaCl salt, respectively, compared to those under non-saline MS media). This finding backs up the hypothesis that high Na^+^ levels damage plants through disturbing protein synthesis (Batool et al. 2014). Additionally, Rasool et al. (2013) and Ahmad et al. (2016) claimed that soluble osmolytes such as proline, soluble proteins, soluble carbohydrates, and glycine betaine help plants deal with overawed osmotic stress caused by salt build-up. However, when *T. viride* was applied under control conditions or when seedlings were exposed to 50 and 100 mM NaCl, respectively, the protein content rose by 11.2, 25, and 23.04% in comparison to the corresponding control seedlings. *T. viride* is essential for plant tolerance; in plants exposed to salinity, proteins act as an energy reserve or perhaps as an osmotic potential regulator (Torabi 2014; Gupta et al. 2020). These observations are consistent with Metwally's (2020) results about *T. viride*-inoculated onion plants. Similar to how Zhang et al. (2016) corroborated our findings by applying *T. longibrachiatum* to wheat seedlings. Moreover, Dief et al. (2021) indicated that protein considerably decreased in the wheat seedlings after NaCl treatment; however, with *Phanerochaete chrysosporium* application its content increased greatly.

On the contrary, both NaCl concentrations cause a conspicuous increase in proline content of tomato seedlings, where its highest value was observed at 100 mM NaCl (Fig. 6c). In harmony with this finding Ueda et al. (2007) and Khomari et al. (2017) stated that plants accumulate compatible osmolytes, such as proline, during salt stress to assist with osmotic adjustment and boost dehydration tolerance. Our findings consistent with the findings of Rawat et al. (2011) and Dief et al. (2021) that seed bio-priming with *T. harzianum* and *P. chrysosporium* increased proline content in wheat seedlings. Moreover, previous studies of Zhang et al. (2016) and Khomari et al. (2017) demonstrated that under control and salt stress conditions, *Trichoderma* sp. and *T. longibrachiatum* significantly affected the proline and protein levels of wheat and soybean. The elevation in proline concentrations may be caused by the increased activity of proline-synthesizing enzymes and decreased activity of catabolizing enzymes, or by its constrained assimilation in protein synthesis (Hashem et al. 2015). As well, the further increase in their contents with *T. viride* indicates that *Trichoderma* may be able to provide systemic resistance to the treated tomato seedlings by up-regulating the molecules capable of producing significant osmotic adjustments as well as energy storage (Rasool et al. 2013) as well as energy storage (Zhang et al. 2016). Additionally, higher proline concentrations improved plants' capacity to detoxify accumulated ROS and safeguard seedlings from oxidative damage (Ahmad et al. 2015; Metwally and Abdelhameed 2019).

As shown in Fig. 6e and f, the total phenolic and flavonoid contents of tomato seedlings were measured as 8.594 mg GAE/g FW (mg of gallic acid equivalents on a basis of FW) and 1.312 mg QE/ g FW (mg of quercetin equivalents on a basis of FW) in the control. However, a noticeable increase in the total phenolic content was noticed at 50 and 100 mM NaCl (Fig. 6e). Similar trend was reported for the flavonoids in tomato seedlings, the accumulation of it was enhanced at both low (50 mM NaCl) and high salt levels (100 mM NaCl). Furthermore, results in (Fig. 6e and f) showed that the treatment of tomato seedlings with *T. viride* (T1, T3 and T5) increased the phenolic and flavonoid contents compared to the control one. These results are in line with those of Alwhibi et al. (2017) and Ikram et al. (2019), who found that under drought and salinity stress, tomato and wheat plants coupled with *T. harzianum* and *T. reesei* had higher levels of flavonoid and phenolic compounds than control plants. The manufactured phenolic and flavonoid molecules exhibit critical roles in many aspects of plant life, including disease resistance, reproduction, growth, and protection from abiotic stress (Alwhibi et al. 2017).

### Salt mitigation induced by *T. viride* is dependent on an enzymatic system

**Fig. 7:**
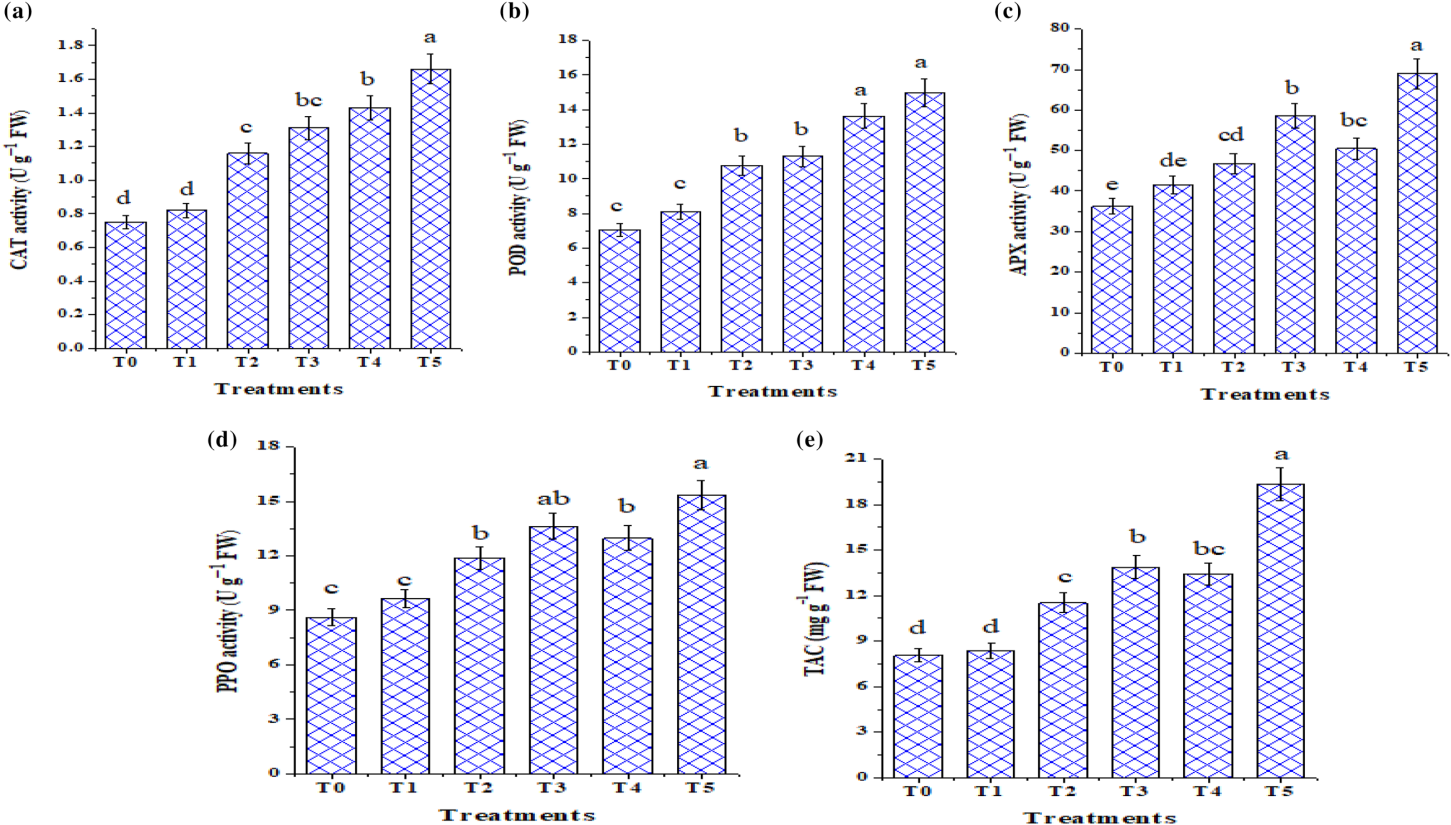
Effect of *T. viride* application on antioxidant enzymes system in tomato seedlings in response to salinity stress. T_0_, T_2_ and T_4_ represent tomato seedlings grown on MS medium under 0, 50 and 100 mM NaCl stress, while T_1_, T_3_ and T_5_ represent tomato seedlings grown on MS medium with *T. viride* under 0, 50 and 100 mM NaCl stress.*Data are the mean of five replicates ± standard error (n = 5). Different letters above bars indicate a significant difference between treatments using ANOVA followed by Duncan’s multiple range test (*p* < 0.05)

We examined the activity of some antioxidant enzymes in tomato seedlings, including POD, PPO, CAT, and APX, as well as total antioxidant capacity (TAC), in an attempt to assess whether the enzymatic system contributes to the estimation of whether *T. viride*-induced tolerance to salt by scavenging ROS (Fig. 7a–e). Results show that all antioxidant enzymes measured increased significantly in response to NaCl stress besides TAC in tomato seedlings (Fig. 7). Furthermore, their activities were significantly increased after *T. viride* treatment under saline and non-saline stress conditions, compared to their corresponding control. Under non-saline MS medium, *T. viride* increases POD, CAT, and APX by 15.03, 9.33, and 14.88%; respectively. Whereas, under 50 mM NaCl it increases these values by 60.2, 74.6, and 61.6% as compared to the control ones. Where, these antioxidant enzymes in tomato seedlings perform the main role in ROS scavenging and hence, reducing the oxidative stress-induced negative effects on several sensitive molecules, such as nucleic acids, proteins, and lipids (Ebrahim and Saleem 2017; Abdelhameed and Metwally 2019). Our findings support Zhang et al. (2016) and Zhang et al. (2019a) in that *T. longibrachiatum* T6 and *T. harzianum* strengthened the antioxidative defense, which improved wheat and cucumber's tolerance to salt stress. Additionally, wheat leaves bio-primed with *P. chrysosporium* and subjected to salt stress showed a remarkable rise in the activity of the CAT and APX enzymes (Dief et al. 2021). These findings suggest that *T. viride* could increase the antioxidant enzyme activities in tomato seedlings to confer systemic resistance and result in higher FW, DW, and seedlings length (Fig. 5) in tomato seedlings.

### Plant growth promotion of *T. viride*-treated tomato under greenhouse conditions

**Fig. 8:**
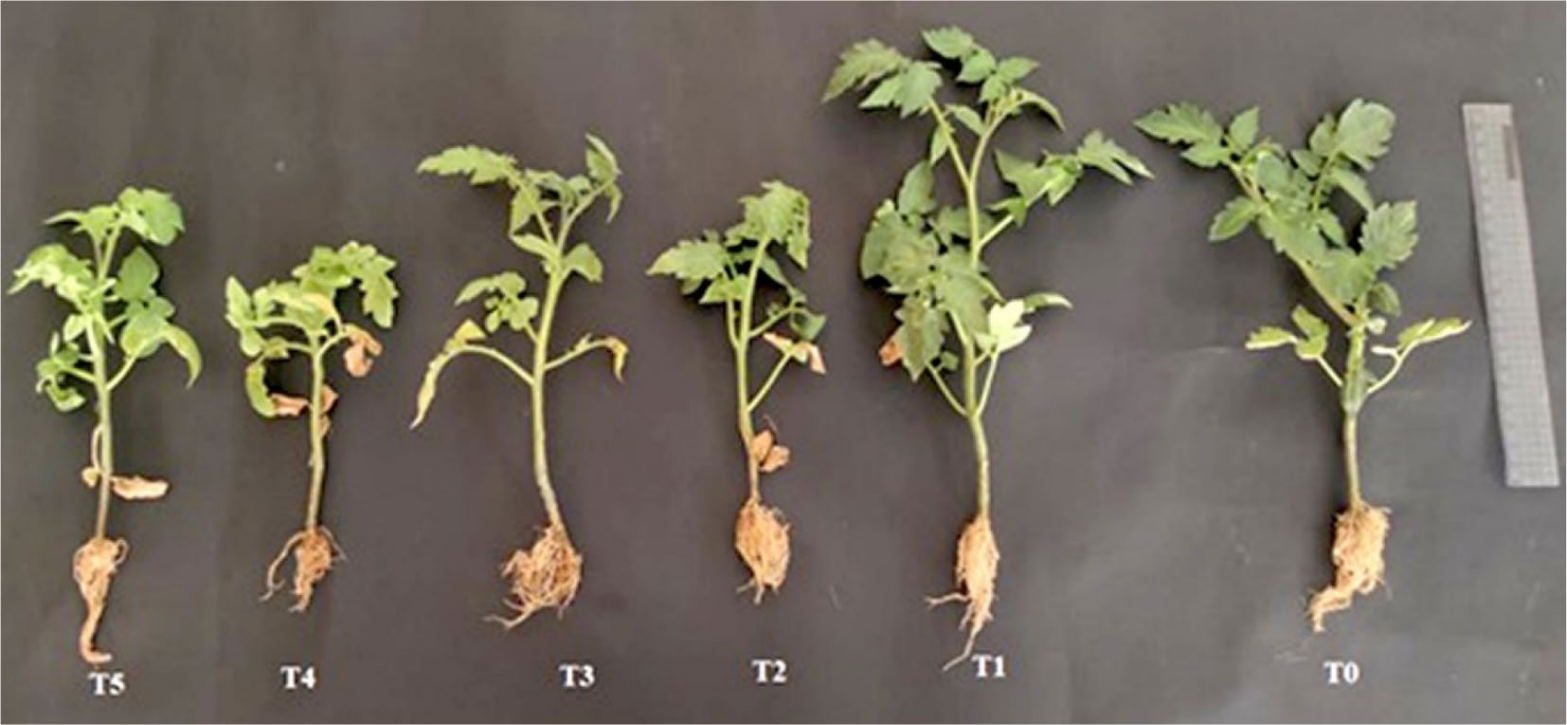
Photographs of tomato plants showing the differences between control and salt stressed plants under *T. viride* fungal application. T_0_, T_2_ and T_4_ represent tomato plants grown under 0, 50 and 100 mM NaCl stress, while T_1_, T_3_ and T_5_ represent tomato plants grown with *T. viride* under 0, 50 and 100 mM NaCl stress

According to the findings listed in Table 5, both salt concentrations exhibited inhibiting effects on the tomato plants' shoot height, root length, FW and DW of shoots and roots, and the number of leaves as compared to the control plant. However, plants not treated with *T. viride* showed a greater reduction in these parameters than plants treated with *T. viride* (Fig. 8). Additionally, the decline was greater at the highest salt concentration (100 mM) than at the lowest (50 mM). According to Metwally and Abdelhameed (2018) and Evelin et al. (2019), salt stress has suppressive effects. The non-availability of nutrients and the expenditure of energy to mitigate the detrimental effects of NaCl are potential explanations for the salt inhibitory impact (Siddiqui et al. 2009; Abdel Latef and Chaoxing 2014). *Trichoderma* establishes symbiotic interactions with a wide range of plant roots and promotes their growth and development, regardless of the salt level, *T. viride* application mainly improved tomato growth indices in *T. viride*-applied plants as opposed to non-applied ones (Gupta et al. 2020). Additionally, the ability of Trichoderma to produce IAA and boost ACC-deaminase activity is crucial for the encouragement of tomato growth under salt stress (Zhang et al. 2019b).

**Table 5:** Effects of NaCl stress and *T. viride* on morphological traits of tomato plants under greenhouse conditions

Treatments	Shoot height (cm)	Root length (cm)	Shoot FW (g)	Root FW (g)	Leaves number	Shoot DW (g)	Root DW (g)
T_0_	17.5 ± 0.926b	5.5 ± 0.291c	6.1 ± 0.322b	1.21 ± 0.064b	9.33 ± 0.494a	0.8021 ± 0.042b	0.1666 ± 0.0088b
T_1_	21.5 ± 1.137a	7 ± 0.37a	8.23 ± 0.435a	1.39 ± 0.073a	9.66 ± 0.511a	1.1362 ± 0.061a	0.2407 ± 0.0127a
T_2_	14 ± 0.74c	5.1 ± 0.269c	5.02 ± 0.266cd	0.925 ± 0.049c	7 ± 0.37bc	0.5939 ± 0.031cd	0.1046 ± 0.0055c
T_3_	16 ± 0.85bc	6.5 ± 0.374ab	5.62 ± 0.297bc	1.13 ± 0.059b	8 ± 0.423b	0.6798 ± 0.036c	0.1527 ± 0.008b
T_4_	11 ± 0.58d	4 ± 0.212d	3.68 ± 0.195e	0.538 ± 0.028e	6 ± 0.317c	0.5125 ± 0.027d	0.0785 ± 0.0041d
T_5_	14.5 ± 0.77c	6 ± 0.317bc	4.45 ± 0.235de	0.725 ± 0.038d	7.5 ± 0.396b	0.5867 ± 0.031cd	0.0979 ± 0.0052cd

T_0_, T_2_ and T_4_ represent tomato plants grown under 0, 50 and 100 mM NaCl stress, while T_1_, T_3_ and T_5_ represent tomato plants grown with *T. viride* under 0, 50 and 100 mM NaCl stress

^*^Data are mean of 5 replicates ± standard error

Different letters in the same column mean significant difference at the *p* < 0.05 level by Duncan’s new multiple range test

## Conclusions

From results of our study, it becomes clear that higher salt concentrations significantly affect the colony diameter and DW of *T. viride* as well as the tomato seedlings growth parameters. However, *T. viride* treatment somewhat reduced these negative salinity effects. Additionally, the increased ROS scavenging (CAT, POD, APX, and PPO) and maintaining the osmotic balance (protein and proline contents) of *T. viride* may be the cause of tomato seedlings' enhanced salt tolerance. The use of *T. viride* is therefore proposed as a good solution and can be suggested for use by farmers as an efficient as well as a cost-effective strategy, against salinity.

## Data Availability

All data generated or analyzed during this study are included in this published article.

## Abbreviations

BSA: Bovine serum albumin
CAT: Catalase
DW: Dry weight
FW: Fresh weight
H_2_O_2_: Hydrogen peroxide
MDA: Malondialdehyde
POD: Peroxidase
PPO: Polyphenol oxidase
ROS: Reactive oxygen species
RWC: Relative water content
*T. viride*: *Trichoderma viride*
TBA: Thiobarbituric acid
TCA: Trichloroacetic acid
TW: Turgid weight
WC: Water content
WSD: Water saturation deficient

## Declarations

### Experimental research and field studies on plants

All relevant institutional, national and international guidelines and legislation were complied or adhered to in the production of this study.
